# C. A. Moore

**Published:** 1948

**Authors:** 


### C. A. MOORE, M.S., F.R.C.S.

The recent death of Mr. C. A. Moore on 16th March, 1948, after a long illness, removes an honoured and respected name from the ranks of the medical profession of this city. He was educated at Malvern, and became a student at Bristol University College in 1897.

Moore early showed promise of more than average ability, and he took the "primary fellowship" before commencing his clinical work at the General Hospital. From there he went to the London Hospital for a time, qualifying in 1903. He then did house appointments at the Liverpool Stanley Hospital and the Union Hospital, Sheffield, finally becoming S.R.O. at the Bristol General Hospital in 1908. He was appointed assistant surgeon in 1910, and during the 1914–18 war was in charge of a surgical division at 56 General Hospital, France. In the recent war he was regional advisor in surgery to the Ministry of Health. Moore was due to retire in 1940, but willingly stayed on at the Bristol Royal Hospital and it was during this period that prolonged overwork brought on the illness from which he died. He was a first-rate surgeon, and was noted in his theatre work for punctuality and speed. Showing always a keen sense of humour he had perhaps sometimes a mordant wit, but his popularity with students was evident by the large number that became his dressers.

Clifford Moore had other interests outside his profession. He had a great love for good music, he was enthusiastic over beautiful scenery, a taste which he gratified by many visits to Switzerland and North Wales. Bird-life was another of his interests and, in his early days, he was a keen photographer.

He will long be remembered both with affection and respect by his colleagues, and we tender our very sincere sympathy to his wife, a former theatre sister, and his family.

